# School Satisfaction in Immigrant and Chilean Students: The Role of Prejudice and Cultural Self-Efficacy

**DOI:** 10.3389/fpsyg.2020.613585

**Published:** 2020-12-10

**Authors:** María José Mera-Lemp, Marian Bilbao, Nekane Basabe

**Affiliations:** ^1^Faculty of Psychology, Universidad Alberto Hurtado, Santiago, Chile; ^2^Faculty of Psychology, University of the Basque Country, Donostia-San Sebastián, Spain

**Keywords:** immigrant adolescents, prejudice, cultural self-efficacy, satisfaction with school, intergroup relations

## Abstract

Latin-American immigration has transformed Chilean schools into new multicultural scenarios. Studies about intergroup dynamics among students from different cultural backgrounds and their psychological consequences are still limited in south–south migration contexts. Literature has suggested that intergroup relations influence students’ satisfaction with school, and they could be improved by the development of competences to cope with cultural differences. This study aims to verify if cultural self-efficacy and its dimensions mediated the influence of prejudice on satisfaction with school, in a sample composed by *N* = 690 Chilean and Latin-American immigrant secondary students. Results showed that cultural self-efficacy reduced the effect of prejudice in satisfaction with school, in the cases of both immigrant and Chilean students. The dimensions of cultural self-efficacy in processing information from other cultures and mixing with different others make the difference. Findings’ contributions for the understanding of adolescents’ intergroup relations and psychosocial interventions at school are discussed.

## Introduction

School is the main scenario for intergroup contact between adolescents from different ethnic backgrounds. Quotidian experiences of mixing with different others at school could have important consequences on intergroup attitudes, even affecting their relations with outgroup members on their adulthood (Berry et al., 2006; Abrams and Killen, 2014; Schachner et al., 2018b). Moreover, the quality of the contact between students from different cultural backgrounds has also influence in both their school adjustment and psychological well-being (Berry et al., 2006; Martínez-Taboada et al., 2017; Motti-Stefanidi et al., 2020).

Cultural diversity at schools is a new and challenging reality to the Chilean educational system. During the last years, Chile has shown a progressive increase of the immigrant population, which represents 7.7% of the total inhabitants and mostly came from other Latin-American countries (90.96%) (Instituto Nacional de Estadísticas, 2020). As a consequence, 2.2% of the Chilean student body is composed by immigrant children and adolescents, who are principally enrolled in public (58%) or private subsidized schools (33%) with high levels of economic and social vulnerability (Ministerio de Educación de Chile, 2018).

Studies about immigrant and Chilean students’ intergroup attitudes and school well-being are still limited and have shown diverse results. On the one hand, some researches have suggested the existence of negative intergroup dynamics (Salas et al., 2017; Castillo et al., 2018), while other studies have reported low levels of prejudice among both groups (Mera-Lemp and Martínez-Zelaya, in press). A recent Organization for Economic Cooperation and Development (OECD) report for Chile (Guthrie et al., 2019) also revealed that immigrant students tend to inform higher levels of bullying, lower levels of belonging to school, and fewer well-being than Chileans.

Subjective well-being can be understood as a global evaluation of one’s life and its different circumstances and contexts (Diener et al., 2003). The study of children and adolescents’ subjective well-being has multiple approaches (Ben-Arieh et al., 2014), with the most used measures being the evaluation of their satisfaction with life as a whole and by different domains. The studies about adolescent well-being have increased in the last decades, but there are fewer publications of studies about children and adolescents than about adults (Casas, 2011). This is important given the fact that well-being changes significantly with age (Casas, 2010), so learning more about adolescents’ subjective well-being in particular can be interesting to better understand their development and life experiences.

In addition to age differences, literature also shows contrasts according to gender and group status, among others. For instance, a research in Spain showed that girls report higher levels of satisfaction with several domains of their lives (home, material possessions, relationships, neighborhood, and their school) (Casas et al., 2013). This study also found that immigrant students have lower levels of life satisfaction in almost all domains (but the area they live in) than native students. Besides few studies, research on adolescents’ subjective well-being does not usually take into account characteristics of subgroups, such as immigrants, even though they use samples that include different subpopulations (Ben-Arieh et al., 2014). For this reason, it is necessary to develop more studies that look into differences of their subjective well-being to have a deeper comprehension of the phenomenon.

Social relationships are an important pillar for adolescents’ development and subjective well-being. A meta-analysis about social support and children and adolescents’ well-being (Chu et al., 2010) found a small positive association that gets stronger with the increase in age of participants, age being a significant moderator. Also, among different types of social support, the one with the higher association with adolescents’ well-being was perceived social support [*r* = 0.201, CI (0.199, 0.0204)]. Other studies reported that positive peer and friend relationships are positively associated with most domains of adolescents’ life satisfaction (Casas et al., 2004; Casas and González, 2017) and positive affects (Rodríguez-Fernández et al., 2016).

Adolescents’ social relationships mostly happened with their family and schoolmates, which makes school context an important domain of adolescents’ subjective well-being (Eccles and Roeser, 2011; Huebner et al., 2014). Studies of subjective well-being at school have shown that satisfaction with classmates is highly associated with adolescents’ life satisfaction (Casas et al., 2013; Huebner et al., 2014; Casas and González, 2017) as well as school satisfaction and satisfaction with school experience (Whitley et al., 2012; Casas and González, 2017), classmates’ social support being an important variable for explaining school satisfaction (DeSantis King et al., 2006). Correspondingly, classroom climate is positively associated with school satisfaction (Verkuyten and Thijs, 2002; Benbenishty and Astor, 2005; Zullig et al., 2011), which should be considered in multicultural scenarios. Some studies have reported that girls are more satisfied with school than are boys (Karatzias et al., 2001; Mok and Flynn, 2002; Verkuyten and Thijs, 2002; Casas and González, 2017). Other studies have found that immigrant students also have higher levels of school satisfaction than native ones (Verkuyten and Thijs, 2002), but this could be negatively affected by difficulties at classroom, such as language miscomprehension (Vedder et al., 2005).

Another source of difficulties could be the relationships between immigrant and host society members’ schoolmates, especially the tensions produced by intergroup attitudes, which affect their evaluations about their experiences at school (Berry et al., 2006; Fang et al., 2016; Martínez-Taboada et al., 2017; Thijs et al., 2019). Social relatedness is one of the main developmental needs during adolescence. Peers’ orientation, the search of mates’ acceptance, and the belonging to groups turn out to be critical to well-being. The successful achievement of these tasks could lead to positive emotions or, in the opposite, could generate negative emotional experiences (Erikson, 1968; Ryan and Deci, 2000; Verkuyten and Thijs, 2002; Davidson et al., 2010; Tian et al., 2016; Fuligni, 2019).

On the other hand, adolescence is a life stage in which the development of socio-cognitive skills improves the capabilities to comprehend the social environment. This entails a greater salience of the differences between social groups’ members, increasing status systems’ importance on daily interactions with peers. Both the development of these abilities and the intergroup context facilitate the production of outgroups’ bias, influencing relational dynamics in multicultural settings at school (Rutland and Killen, 2015; Miklikowska, 2018; Albarello et al., 2020).

Prejudice has been defined as the negative attitude toward an outgroup or its members, which encompasses affective, cognitive, and behavioral components (Dovidio et al., 2010; Rojas et al., 2014). Research on students’ prejudices has shown that the extent in which their evaluations about outgroups’ members are negative decreases inter-ethnic acceptance and cross-ethnic friendship (van Zalk and Kerr, 2014; Titzmann et al., 2015). These could lead to discrimination and exclusion dynamics, producing negative affective environments at schools (Özdemir and Stattin, 2014; Benner et al., 2015; Benner and Wang, 2017; Brenick et al., 2019). The perception of schoolmates as being hostile or antagonistic, the involvement of negative interactions with peers, and the experience of negative emotions toward them tend to diminish students’ satisfaction with school (Verkuyten and Thijs, 2002; Huebner et al., 2014).

Conversely, literature shows that trusting in peers, establishing positive friendships at school, and receiving prosocial acts from schoolmates improve students’ satisfaction with their lives and also with their schooling processes (Jiang et al., 2013; Huebner et al., 2014; Oyarzun et al., 2017; Su et al., 2019; Varela et al., 2019). These could be especially important for immigrant students’, for whom social relationships at school have also a great role in the promotion of their positive integration to the host country (Verkuyten and Thijs, 2002; Berry et al., 2006; Asendorpf and Motti-Stefanidi, 2017; Schachner et al., 2018a).

Research about intergroup attitudes has also suggested that prejudice could be related to sociodemographic variables, such as sex and age. There is strong evidence (Dozo, 2015) supporting that women tend to present lower levels of prejudice than men regarding different outgroups, including studies conducted with adolescents (Mähönen et al., 2011; Güngör and Bornstein, 2013; Nshom and Croucher, 2018). On the other hand, evidence about age’s influence on prejudice across adolescence is not conclusive. While some studies (e.g., Raabe and Beelmann, 2011) have found no developmental trends in adolescence, other researches (van Zalk and Kerr, 2014; Titzmann et al., 2015) have informed that prejudice tends to decrease in late adolescence. Conversely, other studies (Hooghe et al., 2013; Ngwayuh and Croucher, 2017; Nshom and Croucher, 2018) have reported an increase of prejudice through this period, proposing that at the end of adolescence, youngsters could be more prone to perceive outgroups’ members as competitors for material resources, which might lead to higher levels of intergroup threat perception.

Another critical task for adolescents is the construction of a personal sense of mastery (Erikson, 1968; Bandura, 2006; Deci and Ryan, 2012). Self-efficacy has been defined as individuals’ beliefs and confidence in their own capacity to perform a specific behavior to accomplish a particular result (Bandura, 1997). These beliefs play a significant role in socio-cognitive, emotional, and motivational processes, exerting an important influence on adolescents’ positive development (Vecchio et al., 2007). Self-efficacy beliefs have been shown to have a great impact on adolescents’ social relations, facilitating their competences to establish positive interactions in school settings and attenuating the outcomes of negative encounters with peers (Caprara et al., 2004; Vecchio et al., 2007; Titzmann et al., 2015; Turner and Cameron, 2016; Basili et al., 2020).

Likewise, the quality of adolescents’ inter-ethnic relationships is also related to their capabilities to think and behave successfully in intercultural interactions (Briones et al., 2009; Rania et al., 2012). The construction of cultural competences involves the use of socio-cognitive skills to be aware through intercultural contacts, processing and comprehending cultural-based information. Besides the identification and understanding of cultural discrepancies and similarities, cultural competences require the development of positive emotions toward different others, accepting and respecting their cultural identities and abandoning ethnocentrism (Bhawuk et al., 2008). These feelings motivate people to interact with others from different cultures and improve their willingness to use cultural-based information to adjust their own behavior in intercultural contexts (Chen and Starosta, 2000; Spencer-Rodgers and McGovern, 2002; Earley and Ang, 2003; Hammer et al., 2003; Ting-Toomey, 2009; Rania et al., 2012; Rodenborg and Boisen, 2013; Chao et al., 2017).

Cultural self-efficacy is defined as individuals’ beliefs about their capabilities to boost their own motivations, deploy cognitive resources, and reorient their actions in cultural diversity contexts (Briones et al., 2009; Rania et al., 2012). It stimulates intercultural interactions and enables the construction of optimistic expectations and confidence toward intercultural contact. Thus, cultural self-efficacy facilitates individuals’ satisfaction in inter-ethnic encounters (Herrero-Hahn et al., 2019; Lee and Ma, 2019).

Cultural self-efficacy has been scarcely studied in the context of adolescents’ inter-ethnic relations (Schwarzenthal et al., 2019), and most of the researches in this field have focused on adults working in international teams, social services, or teaching settings (e.g., Reichard et al., 2014; Siwatu et al., 2017; Herrero-Hahn et al., 2019; Lee and Ma, 2019).

Studies with adult populations have reported that ethnocentrism and negative attitudes toward people from different ethnic backgrounds negatively affect cultural self-efficacy’s development, because they inhibit interactions and behavioral adjustment in intercultural settings (Kardong-Edgren et al., 2005; Shaffer et al., 2006; Dollwet and Reichard, 2014; Reichard et al., 2014). In contrast, feeling capable of understanding and mixing in contexts of cultural diversity leads people to be open and disposed to involvement in intercultural relations and also to be resilient when facing difficulties in these interactions (Reichard et al., 2014). Besides, the development of these capabilities in adults has been linked with higher levels of well-being in academic and work scenarios (Yang and Chang, 2017; Kotze and Massyn, 2019).

In the case of adolescents in multicultural school settings, there is some evidence which suggests that negative emotions toward peers, such as anxiety, and the orientation to violence are related to a decrease of intercultural competences (Oyeleke et al., 2018; Bagci et al., 2020). A study conducted in Germany by Schwarzenthal et al. (2020) also reported that changes in students’ cultural competences depend on school climate, in terms of the endorsement of positive attitudes toward multiculturality and the promotion of contact and cooperation.

Also, Briones et al. (2009) in a sample of immigrant and native adolescents in Spain found that cultural self-efficacy enhanced cultural integration orientations and diminished marginalization. Likewise, in a study conducted with immigrant and Italian secondary school students, Rania et al. (2012) showed that cultural self-efficacy was positively related with perceived social support between peers. Recently, studies conducted with immigrant and Chilean adolescent samples (Mera-Lemp et al., 2020a, b) reported that cultural self-efficacy was explained by students’ attitudes toward cultural interchange, as well as by the perception of cultural discrepancies between school and family cultures. Besides, in the aforementioned researches, cultural self-efficacy turned out to be a predictor of school satisfaction. A review of literature about life satisfaction in youth immigrants reported that one of the most consistent predictors of life satisfaction was self-efficacy, while perceived discrimination reduced it among several ethnic groups in Europe, America, and Australia (Proctor et al., 2009).

In summary, these antecedents suggest that prejudice against outgroup members could lead to negative attitudes and higher levels of social distance between students, diminishing school satisfaction in multicultural settings. Literature also proposes that, even when prejudice can exist, cultural self-efficacy could increase students’ capabilities to establish positive relationship, including school experiences. Based on these antecedents and the lack of conclusive evidence about these matters in the Chilean context, this study aims to (1) study possible differences between immigrant and Chilean students on cultural self-efficacy and school satisfaction; (2) establish the influence of sex, age, and length of residence in Chile (immigrant students) on the aforementioned variables; (3) verify the relationships between prejudice, cultural self-efficacy, and school satisfaction perceived by Latin-American immigrants and Chilean students; and (4) establish the possible incidence of cultural self-efficacy on the relationship between prejudice and school satisfaction. As a hypothesis, we expect that (1) Chileans students will present lower levels of school satisfaction than immigrants; (2) girls will present higher levels of school satisfaction and lower levels of prejudice than boys; (3) there will be a negative relationship between prejudice and school satisfaction; and (4) cultural self-efficacy will reduce the negative effect of prejudice on school satisfaction.

## Materials and Methods

### Participants

In Chile, the mean of immigrant students’ concentration at schools is 2.2% (Ministerio de Educación de Chile, 2018), and immigrant population is mainly settled in the Metropolitan Region of Santiago (65.2%) (Instituto Nacional de Estadísticas, 2020). Even though this national information was available, there was no particular data for the school level, which impeded the design of a representative sample. Aiming to guarantee intercultural group contact, six schools with high concentrations of immigrant students were selected (2.3–60.5%). The majority of the students were enrolled in schools financed by the State but administrated by private organizations (69.5%), while 30.5% came from public schools, a proportion that is similar to the national matriculation.

The sample was composed of 691 secondary students (44% immigrants and 56% Chilean), with ages between 13 and 19 years old (*M* = 16.02; SD = 1.41). In the case of immigrant students, 54% of them were women, while in the Chilean group, women represented 52%.

Immigrant students were 304, and all of them were born in foreign Latin-American countries, particularly in Peru (53.3%), Venezuela (24.3%), Colombia (10.9%), Bolivia (5.3%), Ecuador (4.3%), and Dominican Republic (2%). The length of residence in Chile varied from 1 to 180 months (*M* = 43.76; SD = 41.58).

### Variables and Instruments

#### Sociodemographic Questionnaire

Students were asked to inform their sex and age. In the case of immigrant students, country of origin and length of residence in Chile were also reported.

#### School Satisfaction

The School Satisfaction Scale (Casas et al., 2013) was applied. This instrument is composed of six Likert-type items, with 11 answer options (0 = completely disagree, 10 = completely agree). This scale asks about different domains of scholarly experience, such as satisfaction with school achievement, learnings, schoolmates, teachers, and school and satisfaction with their lives as students (immigrants: α = 0.83, Ω = 0.80; Chileans: α = 0.83, Ω = 0.84). The confirmatory factor analysis results were satisfactory in the immigrant group: χ^2^(5) = 12.441, *p* < 0.05; CFI = 0.989; TLI = 0.967; SRMR = 0.028; RMSEA = 0.070 [90% CI (0.021, 0.120)]. In the case of Chilean students, the confirmatory factor analysis results were also adequate: χ^2^(5) = 14.153, *p* < 0.05; CFI = 0.990; TLI = 0.971; SRMR = 0.032; RMSEA = 0.069 [90% CI (0.028, 0.113)].

#### Prejudice

The emotional prejudice scale (Navas and Rojas, 2010) was applied. It is composed of 11 Likert scale items (1 = totally disagree, 7 = totally agree), which assess participants’ positive emotions, subtle negative emotions, and traditional negative emotions toward the correspondent group (e.g., sympathy, discomfort, anger). In this study, immigrant students were asked to indicate the extent in which they feel these emotions toward Chilean students (α = 0.77, Ω = 0.80), whereas Chilean students were asked to answer about their feelings toward their immigrant peers (α = 0.76, Ω = 0.80). In the case of immigrant students, the results of the confirmatory factor analysis were satisfactory: χ^2^(35) = 81,628, *p* < 0.001; CFI = 0.968; TLI = 0.950; SRMR = 0.041; RMSEA = 0.066 [90% CI (0.048, 0.085)]. The confirmatory analysis results for the Chilean group were also adequate: χ^2^(34) = 64.516, *p* < 0.001; CFI = 0.984; TLI = 0.975; SRMR = 0.040; RMSEA = 0.048 [90% CI (0.030, 0.066)].

#### Cultural Self-Efficacy

The Cultural Self-efficacy Scale for Adolescents (CSES-A) (Briones et al., 2009) was applied, and participants answered using a 7-point Likert scale (1 = totally incapable; 7 = totally capable). The original scale included 25 items to assess five dimensions of cultural self-efficacy: self-efficacy in processing information about other cultures, self-efficacy in mixing satisfactorily with other cultures, self-efficacy in understanding other ways of life, and self-efficacy to cope with homesickness and in learning and understanding a foreign language. Due to the fact that in this study the sample was composed of Chilean and immigrant students, three dimensions were used: self-efficacy in processing information about other cultures (e.g., “Use the information I have on that culture to understand people from that culture”) (immigrants: α = 0.89, Ω = 0.89; Chileans: α = 0.88, Ω = 0.88), self-efficacy in mixing satisfactorily with other cultures (e.g., “Take part in social activities of the people of that culture”) (immigrants: α = 0.85, Ω = 0.86; Chileans: α = 0.91, Ω = 0.91), and self-efficacy in understanding other ways of life (e.g., “Understand other religious beliefs”) (immigrants: α = 0.89, Ω = 0.90; Chileans: α = 0.89, Ω = 0.88). In the case of immigrant students, the results of the confirmatory factor analysis were satisfactory, χ^2^(181) = 420.192, *p* < 0.001; CFI = 0.940; TLI = 0.930; SRMR = 0.024; RMSEA = 0.066 [90% CI (0.058, 0.074)], as well on the Chilean student group, χ^2^(181) = 262.780, *p* < 0.001; CFI = 0.973; TLI = 0.967; SRMR = 0.036; RMSEA = 0.053 [90% CI (0.044, 0.062)].

### Procedure

Santiago is a city with a higher concentration of immigrant population in the country (65.2%) (Instituto Nacional de Estadísticas, 2020). Thus, the study was carried out in this city. The communes of the city were studied in order to find the ones with higher immigrant population. Five communes were contacted, in which immigrant population varied from 15.6 to 31.2%. Three of them accepted to participate, and all schools with high immigrant concentrations were invited. Six of them were selected according to their characteristics, such as matriculation size and ethnic composition. These characteristics were studied in order to verify if they had influence on the studied variables, but no significant differences were found.

Participants’ selection was conducted using a convenience sampling, according to schools’ availability to participate. Second-generation immigrant students and the ones with low levels of proficiency in Spanish were excluded. Parents’ and students’ consent were requested by means of personalized letters, aiming to safeguard voluntary participation. Participants completed the questionnaire protocol voluntarily in their educational centers and during regular class time. Questionnaire administration was done collectively and lasted 40 min. The procedures followed in the study were certified by the Research Ethics Committee of Universidad Alberto Hurtado, considering all the standards of the Helsinki Declaration.

### Data Analysis

Data analyses were conducted using the statistical software SPSS 23.0. First, reliability analyses were performed, the results of which have been presented in the preceding section. Kolmogorov–Smirnov test was performed, with the Lilliefors correction, for each of the variables, and the non-normality of the data distribution in some of them was determined. For this reason, correlations were performed by the bootstrap resampling method, with 1,000 samples, and the BCa method to obtain 95% confidence intervals for the *r* (Field, 2013). The effect of the percentage of immigrant students by classroom on the studied variables was not significant. Therefore, this variable was not included in the subsequent analysis. In the case of immigrant students, we also performed comparisons between national groups. Venezuelan and Colombian participants were grouped in the same first category, because they were recent immigrants who have similar migratory backgrounds (*n* = 107). The second group was composed by Peruvians (*n* = 162), who belong to the largest and former immigrant group in Chile. The third group was mixed, and it included students from different minority nationalities, such as Ecuadorians, Dominicans, and Bolivians (*n* = 35). ANOVA test reported no significant differences among groups in the studied variables. Thus, immigrant participants were considered as a single group for the following analysis. Mediation analyses were carried out based on the Process macro (model 4) system of SPSS, which on the bases of the Sobel test establishes whether the indirect effect of the mediator is significantly different from zero. When the confidence interval does not include 0, the effect is significant, and mediation is confirmed (Hayes, 2018). Due to the fact that Chilean and immigrant students presented different levels of school satisfaction and cultural self-efficacy, it was interesting to perform correlations for each group. Even though we used the same prejudice scale in both groups, the target of prejudice was different. We were interested in understanding how the relationships in these variables were expressed in each group. Thus, we carried out different mediational models for immigrant and Chilean participants.

## Results

First, differences among immigrant and Chilean students on the variables of interest were also studied. Even though it was not possible to compare immigrant and Chilean students’ levels of prejudice since scales encompassed different targets, results showed a common pattern with scores under the midpoint on the scale of 7 points. Cultural self-efficacy’s general perceptions tended to be high in both immigrants and Chilean students, and there were no significant differences between groups. The same occurred in the dimensions of mixing satisfactorily with other cultures and self-efficacy in understanding other ways of life. Scores in the dimension of self-efficacy in processing information from other cultures were also over the midpoint on the scale of 7 points in both groups. However, mean comparisons in self-efficacy showed significant differences, in which immigrant students reported higher levels on this dimension, with a small effect size. Scores in school satisfaction were also over the midpoint of the 11-point scale, where immigrant students informed significantly higher levels than Chileans, with a moderate effect size (see Table 1).

**TABLE 1:** Descriptive statistics and mean comparisons between immigrant and Chilean students.

		M	SD	*t*	df	*p*	Cohen’s *d*
Prejudice	Immigrants	2.62	0.88	–	–	–	–
	Chileans	2.08	0.76				
Cultural self-efficacy	Immigrants	5.70	0.99	−0.578	689	0.563	ns
	Chileans	5.65	1.04				
SE processing information	Immigrants	5.85	1.16	−5.551	689	0.000	−0.416
	Chileans	5.34	1.25				
SE mixing with other cultures	Immigrants	5.81	1.02	−0.267	689	0.790	ns
	Chileans	5.78	1.17				
SE understanding different ways of life	Immigrants	5.71	1.24	0.355	689	0.723	ns
	Chileans	5.74	1.30				
School satisfaction	Immigrants	7.44	1.65	−3.550	689	0.000	−0.269
	Chileans	6.97	1.78				

*Prejudice and self-efficacy variables ranged from 1 to 7 and school satisfaction from 0 to 10.*

Separate Pearson’s correlation analyses were performed, due to prejudice having different targets and because it was interesting to observe the particularities of each sample.

In the case of immigrant students, results showed that neither age nor the length of residence in Chile was related to any of the studied variables. Sex was positively associated with both school satisfaction and self-efficacy in mixing satisfactorily with others, suggesting that women were more satisfied with their experiences at school and that they felt more capable of mixing with people of different cultural backgrounds than are men. In this group of students, prejudice was negatively related to school satisfaction, general cultural self-efficacy, and specifically to the dimensions of processing information about other cultures, mixing satisfactorily with others, and understanding other ways of life. As was expected, school satisfaction was positively associated with general cultural self-efficacy, self-efficacy in processing information about other cultures, mixing satisfactorily with other cultures, and understanding other ways of life (see Table 2).

**TABLE 2:** Pearson’s correlations between length of residency, sex, age, prejudice, cultural self-efficacy, and school satisfaction in immigrant and Chilean students between parentheses.

	1	2	3	4	5	6	7	8	9
1	–								
2	0.012	–							
3	0.62	−0.020(−0.047)	–						
4	0.001	−0.009(−0.112*)	−0.008(0.128*)	–					
5	–0.071	0.184**(0.078)	−0.017(−0.038)	−0.243***(−0.170**)	–				
6	–0.039	0.121 (0.041)	−0.022(−0.081)	−0.218**(−0.378***)	0.411***(0.374***)	–			
7	–0.020	0.120 (0.021)	−0.043(−0.095)	−0.196**(−0.337***)	0.397***(0.330***)	0.866***(0.807***)	–		
8	–0.010	0.144*(−0.003)	−0.011(−0.058)	−0.214***(−0.276***)	0.435***(0.350***)	0.863***(0.902***)	0.881***(0.558***)	–	
9	–0.070	0.089 (0.102)	0.028(−0.060)	−0.217***(−0.376***)	0.284***(0.262***)	0.810***(0.825***)	0.529***(0.533***)	0.604***(0.610***)	–

1, length of residency in Chile; 2, sex; 3, age; 4, prejudice; 5, school satisfaction; 6, cultural self-efficacy; 7, SE processing information; 8, SE mixing with other cultures; 9, SE understanding different ways of life. Correlations performed by the bootstrap resampling method, with 1,000 samples, and the BCa method to obtain 95% confidence intervals for the r. * p < 0.05, ** p < 0.01, *** p < 0.001.

In the case of Chilean students, age was positively correlated with prejudice. Sex was negatively associated to prejudice, showing that women tend to perceive less negative emotions toward immigrant peers than did men. Besides, prejudice was negatively related to school satisfaction, as well as to general cultural self-efficacy. Results also showed that prejudice presented negative relations with processing information about other cultures, mixing satisfactorily with other cultures, and understanding other ways of life. School satisfaction was positively associated with general cultural self-efficacy, and its relations with processing information about other cultures, self-efficacy in mixing satisfactorily with other cultures, and understanding other ways of life were also positive (see Table 2).

**FIGURE 1:**
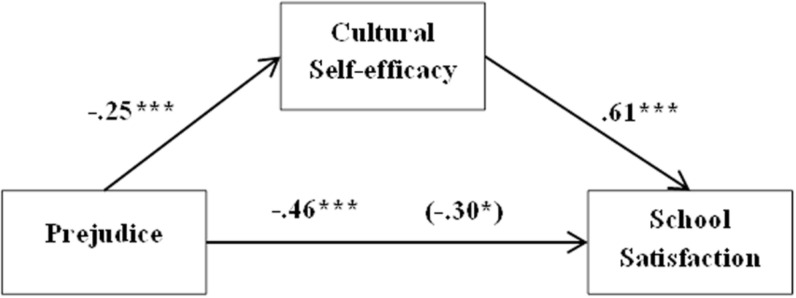
Simple mediation model. Cultural self-efficacy’s effect on the relationship between prejudice and school satisfaction in immigrant students. *p < 0.05, **p < 0.01, ***p < 0.001.

Based on these results, four mediation models were performed to test if cultural self-efficacy mediated the relation between prejudice and school satisfaction in each group of participants. The first model attempted to assess the aforementioned relations on the immigrant students’ group. Age and length of residence in Chile were not included because they did not present significant correlations with any of the studied variables. When the effect of sex (β = 0.57, *t* = 3.129, *p* = 0.002) was controlled for, prejudice had a significant negative effect on school satisfaction (β = −0.46, *t* = −4.932, *p* = 0.000). Prejudice had also a negative influence on cultural self-efficacy (β = −0.25, *t* = −3.89, *p* = 0.000). Conversely, cultural self-efficacy had a positive impact on school satisfaction (β = 0.61, *t* = 6.927, *p* = 0.000). The indirect effect of prejudice on school satisfaction through cultural self-efficacy was significant [β = −0.15, SE = 0.09, 95% CI (−0.2361, −0.0764)]. The direct effect of prejudice in school satisfaction was still significant when all the variables were in the model (β = −0.30, *t* = −3.09, *p* = 0.002). This indicates that cultural self-efficacy partially mediates the relation between prejudice and school satisfaction. The model explained 22% of school satisfaction’s variability (see Figure 1).

Aiming to further comprehend the mediator role of cultural self-efficacy, a second model which included its three dimensions was performed. When the effects of sex (β = 0.57, *t* = 3.129, *p* = 0.002) were controlled for, prejudice had a negative effect on school satisfaction (β = −0.46, *t* = −4.392, *p* = 0.000). Likewise, prejudice had a negative impact in self-efficacy in processing information about other cultures (β = −0.26, *t* = −3.49, *p* = 0.000), in self-efficacy in mixing satisfactorily with other cultures (β = −0.25, *t* = −3.84, *p* = 0.000), and in self-efficacy in understanding other ways of life (β = −0.30, *t* = −3.83, *p* = 0.000). Besides, self-efficacy in mixing satisfactorily with other cultures (β = 0.53, *t* = 2.85, *p* = 0.005) had a positive effect on school satisfaction, while the dimensions of processing information (β = 0.08, *t* = 0.55, *p* = 0.58) and understanding other lifestyles (β = 0.02, *t* = 0.25, *p* = 0.80) were not significant mediators.

**FIGURE 2:**
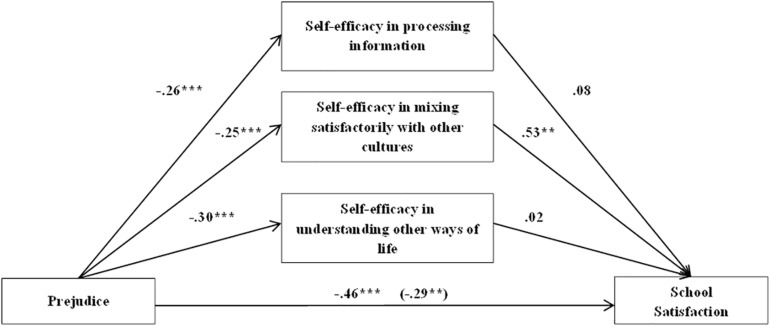
Multiple mediation model. Cultural self-efficacy’s dimensions effects on the relationship between prejudice and school satisfaction in immigrant students. *p < 0.05, **p < 0.01, ***p < 0.001.

A significant indirect effect of prejudice on school satisfaction through self-efficacy in mixing satisfactorily with other cultures was found [β = −0.13, SE = 0.09, 95% CI (−4.6010, −0.0466)]. The direct effect of prejudice was still significant when all the variables were in the model (β = −0.29, *t* = −2.98, *p* = 0.003). Thus, the effect of self-efficacy in mixing satisfactorily with other cultures partially mediated the association between prejudice and school satisfaction. This model explained 23% of immigrant students’ satisfaction with school (see Figure 2).

**FIGURE 3:**
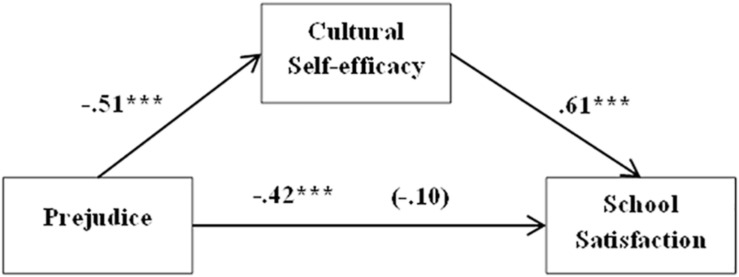
Simple mediation model. Cultural self-efficacy’s effect on the relationship between prejudice and school satisfaction in Chilean students. *p < 0.05, **p < 0.01, ***p < 0.001.

On the other hand, the same models were tested on the Chilean students’ group. In the third model, when the effects of sex (β = −0.35, *t* = −1.967, *p* = 0.954) and age (β = −0.03, *t* = −0.69, *p* = 0.498) were controlled for, prejudice’s negative impact on school satisfaction was significant (β = −0.42, *t* = −0.35, *p* = 0.000). Besides, it had a significant negative influence on cultural self-efficacy (β = −0.51, *t* = −7.080, *p* = 0.000). Cultural self-efficacy had a positive effect on school satisfaction (β = 0.61, *t* = 7.057, *p* = 0.000). The indirect effect of prejudice on school satisfaction through self-efficacy was also significant [β = −0.31, SE = 0.07, 95% CI (−0.4543, −0.1938)], but its direct effect was not significant when all the variables were in the model (β = −0.10, *t* = −0.12, *p* = 0.395). These results indicate that cultural self-efficacy totally mediates the relationship between prejudice and school satisfaction. The model explained 15% of the variability of satisfaction with school on Chilean students (see Figure 3).

The fourth model showed that when the possible effects of sex (β = −0.35, *t* = −1.96, *p* = 0.049) and age (β = −0.02, *t* = −0.388, *p* = 0.698) were controlled for, prejudice exerted a significant negative influence on school satisfaction (β = −0.42, *t* = −3.51, *p* = 0.000). It also had a negative effect on self-efficacy in processing information about other cultures (β = −0.54, *t* = −6.81, *p* = 0.000), on self-efficacy in mixing satisfactorily with other cultures (β = −0.42, *t* = −5.55, *p* = 0.000), and on self-efficacy in understanding other ways of life (β = −0.63, *t* = −7.67, *p* = 0.000). Both cultural self-efficacy in processing information from other cultural groups (β = 0.24, *t* = 2.79, p = 0.005) and in mixing satisfactorily with other cultures (β = 0.33, *t* = 3.34, *p* = 0.001) had a positive impact on school satisfaction, but the dimension of understanding other lifestyles was not a significant mediator (β = 0.04, *t* = 4.39, *p* = 0.66).

**FIGURE 4:**
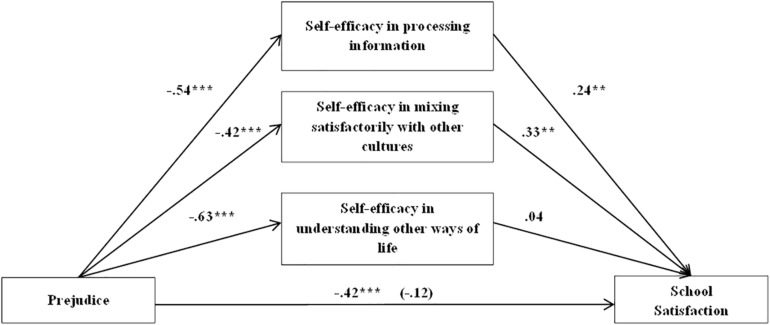
Multiple mediation model. Cultural self-efficacy’s dimensions effects on the relationship between prejudice and school satisfaction in Chilean students. *p < 0.05, **p < 0.01, ***p < 0.001.

The indirect effects of prejudice on the dependent variable through both self-efficacy in processing information [β = −0.13, SE = 0.06, 95% CI (−0.2605, −0.0305)] and self-efficacy in mixing satisfactorily with other cultures [β = −0.14, SE = 0.05, 95% CI (−0.2430, −0.0496)] were significant. The direct effect of prejudice was not significant when all the variables were in the model (β = −0.12, *t* = −0.98, *p* = 0.325). This result shows that these two dimensions of cultural self-efficacy totally mediated the effects of prejudice in school satisfaction. The total amount of explained variability was 17% (see Figure 4).

## Discussion

Intergroup relations have been identified as important antecedents of students’ school adjustment and well-being (Berry et al., 2006; Martínez-Taboada et al., 2017; Motti-Stefanidi et al., 2020; Sirlopú and Renger, 2020). Literature about intergroup contact between immigrant and Chilean students has reported contradictory results. Some researches have informed hostile and conflictive interactions (Salas et al., 2017; Castillo et al., 2018; Guthrie et al., 2019), while others have reported a tendency of low prejudice between students (Mera-Lemp and Martínez-Zelaya, in press). In our research, participants from both groups showed low levels of prejudice, suggesting that intergroup contact tends to be positive in our context. These different findings could be due to the use of different instruments in each study, which have included peer sociometric measures, implicit association test, and explicit prejudice scales.

Besides, students reported high levels of management in cultural diversity scenarios at school, feeling capable to process information from other cultures, mixing satisfactorily with other cultures, and understanding other ways of life. These results coincide with those informed by Rania et al. (2012) and also with previous findings from studies conducted with immigrants and natives in Chile (Mera-Lemp et al., 2020a, b).

In this context, immigrant students tended to show higher self-efficacy than did Chilean in processing information from other cultures, which is also consistent with former reports (Rania et al., 2012). Literature on self-efficacy stresses the importance of past experiences in constructing self-confidence in overcoming a task. Also, vicarious experiences through similar others’ successful performances contribute to self-efficacy development (Bandura, 1996, 2006). Thus, this result suggests that in the case of immigrant adolescents, the daily exercise of managing information from a new cultural environment could activate their cognitive resources, enhancing their personal sense of capability. The observation of positive performances in their families and communities could also play a role on the increment of their perception of self-efficacy.

Participants from both groups also informed levels of school satisfaction over the midpoint of the scale. Different to previous research findings (Guthrie et al., 2019), immigrant adolescents in our study presented a significantly higher school satisfaction than their Chilean peers. This result is coherent with several studies (Verkuyten and Thijs, 2002; Sam et al., 2008; Dimitrova et al., 2016, 2017; Salmela-Aro et al., 2018) that have empirically supported the idea of the immigrant paradox, showing that young immigrants presented similar or better adaptation levels than their native peers. The strong educational aspirations of immigrant parents, the development of bicultural identities, and resilience have been proposed as variables that could be explaining this phenomenon (Dimitrova et al., 2016, 2017; Özdemir and Özdemir, 2020). Nevertheless, this might be deeply investigated in future researches. Overall, these results suggest that intergroup dynamics at schools tend to be positive. Furthermore, both immigrant and Chilean students show important psychological assets which could be collaborating with their adjustment to cultural diversity at school.

In the case of immigrant students, we found that age and length of residence in Chile were not related to any of the studied variables. An interpretation could be that social and cultural conditions might be more decisive than time in their adjustment to the new context (Raabe and Beelmann, 2011; Hernando et al., 2013; Titzmann et al., 2015). Results also showed that immigrant girls presented higher levels of self-efficacy in mixing satisfactorily with other cultures, which coincides with previous reports (Berry et al., 2006; Motti-Stefanidi et al., 2008; Güngör and Bornstein, 2013; Klein et al., 2020) that have claimed that girls are more likely to develop positive attitudes toward intercultural exchanges than boys, presenting higher self-efficacy in social interactions with different others (Caprara et al., 2004; Bagci, 2018). Gender differences were also found regarding immigrant students’ school satisfaction, agreeing with studies which have established that girls tend to develop a better adjustment to school contexts and tasks than did boys, including immigrant females (Verkuyten and Thijs, 2002; Berry et al., 2006; Liu et al., 2016).

Outcomes from the Chilean students’ group showed that the increment of age was related to an increase in prejudice. Reports from former studies about this relation during adolescence are diverse. For example, a meta-analytical research (Raabe and Beelmann, 2011) concluded that prejudice varied systematically through childhood, whereas no developmental trend was found in adolescence. Other studies (van Zalk and Kerr, 2014; Titzmann et al., 2015) have reported a reduction of prejudice on late adolescence. To the contrary, our finding supports other research outcomes which found an increment of prejudice across this period of life (Hooghe et al., 2013; Ngwayuh and Croucher, 2017; Nshom and Croucher, 2018). The aforementioned studies have proposed that during the transition to adulthood, native adolescents tend to perceive increasing levels of intergroup threats, being more sensitive and concerned about ingroup interests. This could be especially plausible in the case of students from vulnerable communities, like the participants in our study, who are aware about economic difficulties on their families and could perceive immigrants as real competitors. In contrast, results suggest that this phenomenon is not salient in the case of immigrant adolescents, probably because of the socio-cognitive and the emotional processes needed for their personal adjustment to the multicultural context (Berry et al., 2006).

Also, in the Chilean group, girls presented fewer levels of prejudice than boys. Literature has plenty of evidence about this relationship. A meta-analytical study conducted by Dozo (2015) including 355 studies found that men were more prejudiced than women toward different outgroups. Besides, researches with a focus on immigrant and native adolescents (Mähönen et al., 2011; Güngör and Bornstein, 2013; Nshom and Croucher, 2018) have reported the same tendency, proposing that compared to men, women are less prone toward social dominance and that their perceptions about outgroups tend to be more nuanced.

Correlation analyses between prejudice, cultural self-efficacy, and school satisfaction showed a common pattern across both groups of students. Prejudice was negatively related to school satisfaction, as was expected. This result emphasizes the importance of peer intergroup relationships in students’ well-being, which are especially sensible in cultural diversity scenarios (Verkuyten and Thijs, 2002; Berry et al., 2006; Fang et al., 2016; Martínez-Taboada et al., 2017; Thijs et al., 2019). Prejudice also presented negative associations with cultural self-efficacy and its dimensions in each group of adolescents. This confirms our hypothesis, suggesting that negative emotions toward outgroups’ members diminish the sense of competence in intercultural encounters, by reducing the sensitivity to manage information from different cultures and the confidence in successfully mixing with outgroup schoolmates (Rania et al., 2012; Oyeleke et al., 2018; Bagci et al., 2020). As was expected, positive relations between cultural self-efficacy and school satisfaction were found. Important aspects of school life are grades and other accomplishments (Casas et al., 2013) that are related to self-efficacy. In the context of these participants, the sense of successfully managing in a multicultural classroom can give them this feeling of competence (Vecchio et al., 2007; Mera-Lemp et al., 2020a).

Simple mediation analyses suggest that the role of cultural self-efficacy in the relation between prejudice and school satisfaction works slightly different for each group of students. Cultural self-efficacy partially reduced the impact of prejudice on immigrants’ school satisfaction, while its effect in the case of Chilean students was total. A plausible explanation for these outcomes could be found in studies which have evaluated results of intervention programs to improve intergroup relations between majority and minority groups’ students. These studies have suggested that their impacts tend to be stronger on the first ones, because those who are members of low-status groups still have to cope with social and cultural barriers (Beelmann and Heinemann, 2014; Titzmann et al., 2015; Cameron and Turner, 2017).

Multiple mediation analyses results including cultural self-efficacy’s dimensions offer a deeper comprehension about these phenomena. In the case of immigrant students, cultural self-efficacy in mixing satisfactorily with other cultures was the only significant mediator, stressing the importance of social relatedness to school experiences and especially to well-being (Casas et al., 2013). The sense of self-mastery in this task seems to be particularly salient in this group of adolescents, who often have to deal with different migration stressors, such as loneliness, discrimination, and separation from their former support networks, and for whom school is a critical scenario for their integration to the host country (Berry et al., 2006; Mera-Lemp et al., 2014). This outcome also suggests that for immigrant students, self-efficacy in processing information from other cultures and in understanding other ways of life could have a less decisive role in reducing the negative influence of prejudice toward schoolmates in school satisfaction, probably because they have to deal continuously with cultural differences through their daily lives.

On the other hand, results from the Chilean group revealed that cultural self-efficacy both in processing information from other cultures and in mixing with other cultures totally mediated the effect of prejudice on school satisfaction. Literature on cultural intelligence (Bhawuk et al., 2008; Alexandra, 2018) has proposed that success in processing information through intercultural encounters depends on complex thinking processes, which require a change in their own cultural baselines. This could be especially challenging for adolescents of majority groups, who have been socialized in monocultural environments, with scarce previous opportunities of intercultural contact. So when contact occurs in positive conditions, it has more impact on majority members’ intercultural skills than in minority ones (Kim and Van Dyne, 2012; Alexandra, 2018). Taking this as a whole, these findings show that feeling capable of interacting with different others improves native students’ well-being, who also have to adjust to a new cultural scenario at school.

It is also interesting that for immigrant and native students, cultural self-efficacy in understanding different ways of life was not a significant mediator in the relationship between prejudice and school satisfaction. We think that this may be due to the aspects that this dimension assessed. Cross-cultural similarities between Latin-American countries in family configurations, religion, and artistic expressions (Medina Nuñez, 2019) could explain the low importance of self-competence sense in comprehending these aspects to school well-being.

This study presents limitations to be considered. The use of an intentional sample in the framework of a cross-sectional design does not allow appreciation of the development of the relationship between prejudice, cultural self-efficacy, and school satisfaction across time. This could be important on this population due to the changes that occur during adolescence. Second, in this research, we did not include immigrant students who could perceive higher levels of cultural differences and also have to deal with language barriers at school, like in the case of Haitian students. They were excluded because they represented a small percentage of the sample. The results of this study could vary by including this type of students due to stronger difficulties to cope with school relationships and tasks (Vedder et al., 2005). Besides, all foreign students in our sample were first-generation immigrants, who were dealing with cultural adjustment processes. Future researches might incorporate second-generation immigrant students in order to study if their outgroup attitudes and psychological assets to cope with cultural differences vary. Also, in this study, the dimension of self-efficacy to cope with homesickness was not included in order to match the surveys; thus, it will be interesting to include this dimension in studies with immigrant students. In addition, a future challenge could be to achieve representative samples and to perform nested models.

However, our results stress the importance of intergroup relations and cultural self-efficacy in school satisfaction. Furthermore, our findings suggest a favorable context, which could be less conflictive than was expected. This could be due to the nuances of south–south migration processes (Mera-Lemp et al., in press), different than traditional south–north migration studies. This is important because researches about intergroup relations between immigrant and native students in Latin-American countries are still very scarce, and most of the evidence about adolescents’ intercultural competences comes from different cultural contexts.

Finally, we think that multicultural education programs in our region should include the development of students’ cultural self-efficacy. These are self-beliefs and skills that can be learned, which will improve adolescents’ well-being in diversity contexts and help the construction of cultural integration and social cohesion. Multicultural education to promote the creation of a culture of peace is one of the main objectives of UNESCO for Latin America and the Caribbean. The development of interventions through which students could learn capabilities to understand cultural differences and mix with different others might facilitate respect and friendship between people with different national, ethnic, and religious identities (García-Ruiz, 2010). The attainment of these changes requires the incorporation of these matters on school curriculums. To achieve these goals, it would be important for teachers to be trained in these subjects through their professional formation. Educational policy about multicultural education should consider the impact of migration on the construction of new types of social relationships between children and adolescents.

## Data Availability Statement

The datasets generated for this study are available on request to the corresponding author.

## Ethics Statement

The studies involving human participants were reviewed and approved by the Comité de ética Universidad Alberto Hurtado. Written informed consent to participate in this study was provided by the participants’ legal guardian/next of kin.

## Author Contributions

MM-L, MB, and NB designed the study. MM-L collected the data. All authors contributed in the analysis and interpretation of data, drafted the manuscript, discussed the results, and commented on the manuscript.

## Conflict of Interest

The authors declare that the research was conducted in the absence of any commercial or financial relationships that could be construed as a potential conflict of interest.
